# Comparison of Clinical Outcomes Between Alpha-Stat and pH-Stat Strategies During Hypothermic Circulatory Arrest: A Systematic Review

**DOI:** 10.7759/cureus.87279

**Published:** 2025-07-04

**Authors:** Georgia Chatzopoulou, Christos Voucharas, Ivana Kougkouli, Stamatis Stathoulopoulos, Vasiliki Linardatou

**Affiliations:** 1 Cardiothoracic Surgery, Aristotle University of Thessaloniki, Thessaloniki, GRC; 2 Intensive Care Unit, General Hospital of Thoracic Diseases “Sotiria” Athens, Athens, GRC; 3 Intensive Care Unit, Hippokration General Hospital of Athens, Athens, GRC; 4 Nursing Education Office, Emergency and Critical Care Nursing, General Hospital of Athens “G. Gennimatas”, Athens, GRC

**Keywords:** alpha-stat, aortic, arch surgery, cerebral perfusion, circulatory arrest, deep hypothermia, moderate hypothermia, ph-stat

## Abstract

Due to the conflicting effects on cerebral perfusion and metabolic control, the optimal acid-base management strategy for hypothermic circulatory arrest remains a matter of ongoing debate, particularly concerning the α-stat and pH-stat approaches. This article presents a systematic review and analysis of the clinical and physiological effectiveness of these two methods in patients undergoing surgery of the aortic arch or ascending aorta. A comprehensive literature search was conducted across PubMed, the Cochrane Library, CINAHL, and ScienceDirect, covering studies published between 2010 and 2025, in accordance with the Preferred Reporting Items for Systematic Reviews and Meta-Analyses 2020 guidelines. Seven studies meeting the inclusion criteria were analyzed, encompassing both pediatric and adult populations. The primary outcomes assessed were cerebral blood flow, oxygenation indices (SjvO₂, CEO₂), cerebral metabolic rate of oxygen, and postoperative neurological recovery. The evidence indicated that pH-stat management led to a more rapid attainment of target hypothermia and enhanced cerebral oxygen delivery in pediatric patients, largely due to CO₂-mediated vasodilation and more uniform cooling. However, these benefits were accompanied by a higher risk of impaired cerebral autoregulation. In contrast, the α-stat strategy preserved physiologic autoregulatory responses, maintained metabolic stability, and was associated with lower postoperative neurological deficits in adult cohorts. Several of the included studies performed quantitative synthesis or meta-analytical comparison, with a trend favoring α-stat in adult patients regarding long-term neurological outcomes. Although pH-stat may offer specific advantages in neonates and young children, α-stat appears to provide a more physiologically appropriate regulation of pH and cerebral hemodynamics in adults. The heterogeneity of study populations, surgical contexts, and outcome measures reinforces the need for an individualized approach. These findings support the consideration of hybrid strategies employing pH-stat during the cooling phase and α-stat during rewarming to balance perfusion benefits and metabolic control. Ultimately, no single strategy demonstrated consistent superiority, and acid-base therapy should be tailored to factors such as patient age, cerebral vulnerability, and procedural complexity.

## Introduction and background

Hypothermic circulatory arrest (HCA) is a proven method for cerebral protection and a bloodless operating field in the management of complex aortic operations [1]. In HCA, acid-base control is crucial secondary to the metabolic shifts under hypothermia and its effect on cerebral perfusion and neurological recovery. Two major approaches have been used clinically, namely, the α-stat and the pH-stat method.

The α-stat approach preserves the patient’s naturally occurring, temperature-induced pH alterations while maintaining intracellular enzyme function and cerebral autoregulation. This contrasts with the pH-stat method, which artificially adjusts arterial pH to 7.40 by administering CO₂, thereby increasing cerebral blood flow (CBF) via vasodilation and promoting homogenous cooling, particularly beneficial in pediatric patients [2].

The current theoretical separation of these two approaches emerged in the 1970s and 1980s based on experimental studies in neurophysiology and cardiac surgery. Dr. John Merkin and colleagues [3,4] were among the first to clarify the functional role of temperature correction of pH values on CBF dynamics and enzymatic kinetics, as demonstrated in later comparative studies [1,2].

However, no agreement has been reached regarding which strategy provides superior neurological protection across all patient populations. This systematic review aims to examine the clinical and physiological outcomes of the α-stat and pH-stat approaches as cooling strategies during HCA, focusing on cerebral oxygenation, perfusion, metabolic suppression, and postoperative neurological recovery.

## Review

Methodology

**Figure 1:**
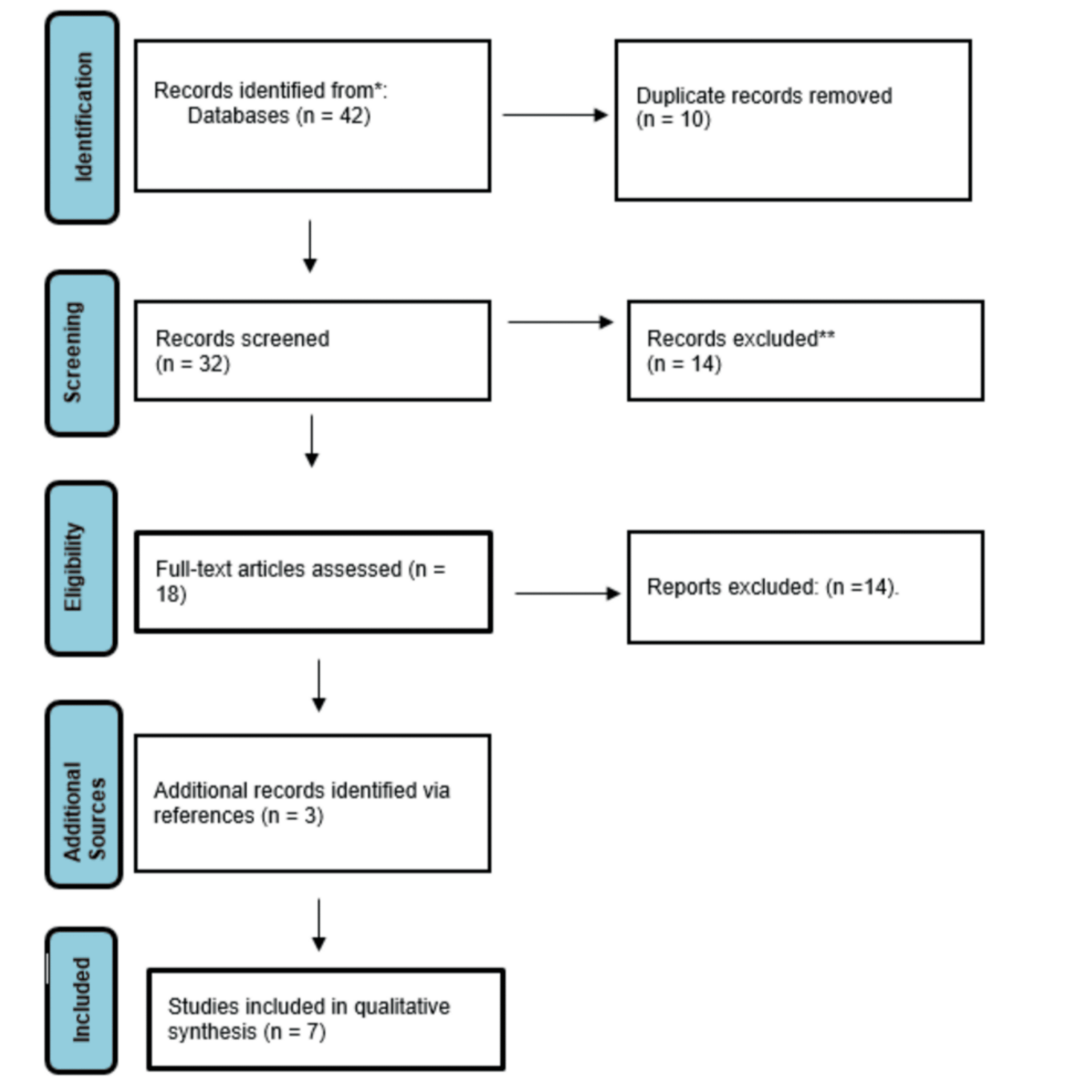
Preferred Reporting Items for Systematic Reviews and Meta-Analyses (PRISMA) flow diagram of study selection. A flow diagram representing the study selection process according to PRISMA guidelines. The figure shows the number of records identified, screened, excluded, and included in the final review (n = 7). Adapted from Page et al. [5].

This systematic review was conducted in accordance with the Preferred Reporting Items for Systematic Reviews and Meta-Analyses (PRISMA) 2020 guidelines [5]. The study selection process is illustrated in the PRISMA flow diagram (Figure 1).

Search Strategy

A comprehensive literature search was performed in four major databases, namely, PubMed, the Cochrane Library, ScienceDirect, and CINAHL, covering studies published between 2010 and 2025. Keywords included aortic, arch surgery, circulatory arrest, cerebral perfusion, pH-stat, alpha-stat, deep hypothermia, and moderate hypothermia. Boolean operators (AND, OR) were used to enhance the sensitivity and comprehensiveness of the search.

Eligibility Criteria

Eligible studies were prospective randomized controlled trials (RCTs) or systematic reviews involving human participants, published in the English language, and directly comparing α-stat and pH-stat strategies during HCA. Only studies reporting outcomes related to cerebral oxygenation, metabolic suppression, or neurocognitive performance were considered. Exclusion criteria included retrospective studies, studies lacking control groups, incomplete or non-peer-reviewed reports, and articles not focused on HCA or acid-base management strategies.

Selection of Studies

The initial search identified 42 records. After the removal of 10 duplicates, 32 unique articles remained for screening. Titles and abstracts were assessed for relevance, leading to the exclusion of 14 studies. The full texts of the remaining 18 articles were reviewed in detail, of which 14 were excluded for not meeting the predefined inclusion criteria. An additional three relevant studies were identified through reference list screening. In total, seven studies fulfilled all eligibility criteria and were included in the final analysis.

Results

A total of seven studies met the predefined inclusion criteria and were incorporated into the final analysis, encompassing both pediatric and adult patient populations (Table 1). These included RCTs conducted across various geographic regions. Table 1 provides a summary of study characteristics and primary findings.

**Table 1:** Comparative summary of the included studies. A summary of key characteristics and findings from the seven included studies comparing α-stat and pH-stat strategies under hypothermic circulatory arrest. Data include study type, sample size, intervention, outcomes, and country of origin. CBF: cerebral blood flow; CPB: cardiopulmonary bypass; DHCA: deep hypothermic circulatory arrest; RCT: randomized controlled trial

Study details	Study type	Patients	Procedure	Findings	Comments	Country
Abdul Aziz and Meduoye [1] (2010)	Prospective RCT	160 (80 α-stat, 80 pH-stat)	α-stat vs. pH-stat under DHCA	pH-stat: faster cooling and better neurological recovery; α-stat: lower morbidity	Large six-month follow-up	UK
Voicu et al. [2] (2014)	Prospective RCT	88 (44 α-stat, 44 pH-stat)	Post-arrest hypothermia	pH-stat: higher CBF; α-stat: lower SjvO₂	Supports pH-stat perfusion	France
Durandy [6] (2010)	Pediatric RCT	<10-year-old children	CPB under hypothermia	pH-stat is ideal for cooling, α-stat for rewarming	Alternating strategy by phase	France
Yu et al. [7] (2023)	Systematic review and meta-analysis	10 RCTs	Thermal strategy in CPB	pH-stat: better perfusion, higher risk; α-stat safer in adults	Suggests a personalized protocol	China
Darocha et al. [8] (2022)	Prospective RCT	120 (60 α-stat, 60 pH-stat)	Refractory cardiac arrest	pH-stat: low PaCO₂; α-stat: stable autoregulation	Strong statistical analysis	Poland
Werner et al. [9] (2024)	Systematic review	≥10 studies	CBF preservation in DHCA	α-stat: better autoregulation, pH-stat: higher CBF with risk	A detailed comparison approach	Austria
Svyatets et al. [10] (2010)	Systematic review	Not reported	DHCA strategy management	Strategic overview of α/pH-stat under DHCA	A clinical strategy guide	USA

Pediatric Population

In pediatric cohorts, Durandy [6] reported that the pH-stat approach facilitated more rapid achievement of target hypothermia and resulted in more uniform cerebral perfusion, particularly during the induction phase of hypothermia. This improved homogeneity of cooling is attributed to CO₂-induced cerebral vasodilation, which enhances global CBF distribution and promotes even temperature gradients within the brain tissue.

The increased oxygen delivery observed with pH-stat management may be particularly advantageous in neonates and infants, who possess immature autoregulatory mechanisms and are more susceptible to ischemic injury under hypothermic conditions. Yu et al. [7], in a meta-analysis of 10 RCTs, confirmed these findings by demonstrating that pH-stat was more effective in achieving target hypothermia and improving oxygenation indices, including jugular venous oxygen saturation (SjvO₂) and cerebral extraction of oxygen (CEO₂).

However, Yu et al. [7] also noted that this advantage comes at a cost, as pH-stat was associated with a higher risk of impaired cerebral autoregulation, which may increase the likelihood of cerebral hyperemia or microembolic events, especially during rewarming phases. Therefore, while pH-stat offers distinct physiological benefits during cooling in pediatric patients, intraoperative monitoring of cerebral hemodynamics remains essential to minimize potential complications associated with excessive vasodilation.

Adult Population

In adult patients, several studies have consistently demonstrated the advantages of α-stat management over pH-stat during HCA. Abdul Aziz and Meduoye [1] reported that α-stat management preserved cerebral autoregulation by maintaining the natural temperature-dependent reduction in pH and minimizing fluctuations in PaCO₂. This stability in cerebral hemodynamics was associated with improved postoperative neurological outcomes. Voicu et al. [2] further observed that while pH-stat increased cerebral oxygen saturation through CO₂-induced vasodilation, it did not translate into better neurological recovery or survival rates in adults, highlighting the complex interplay between oxygen delivery and autoregulatory integrity.

Additionally, Darocha et al. [8] found that patients managed with α-stat exhibited more stable intraoperative CO₂ levels, better metabolic suppression, and reduced incidences of neurological complications. Werner et al. [9] corroborated these findings, reporting a lower incidence of permanent neurological deficits and superior preservation of autoregulatory function under α-stat guidance. These physiological benefits are likely attributable to the closer alignment of α-stat with the brain’s intrinsic metabolic suppression mechanisms during hypothermia, allowing for more controlled reductions in cerebral metabolic rate of oxygen (CMRO₂) without overwhelming cerebral autoregulatory capacity.

Collectively, the available evidence supports α-stat as the preferred acid-base management strategy in adult populations undergoing HCA, particularly due to its ability to maintain cerebral perfusion stability, minimize cerebral hyperemia, and reduce the risk of neurological injury.

Mixed Findings

Mixed results were reported in studies that examined both pediatric and adult populations, highlighting the complex interaction between cerebral autoregulatory capacity, metabolic demands, and acid-base strategy selection. Svyatets et al. [10] emphasized that while pH-stat can improve cerebral oxygenation parameters through vasodilatory effects, its ability to override cerebral autoregulation may paradoxically increase the risk of cerebral hyperemia, microembolic phenomena, and potential reperfusion injury, particularly during the rewarming phase. This physiologic imbalance may be more pronounced in adult patients who possess more developed, yet potentially more vulnerable, cerebrovascular autoregulatory systems.

Conversely, Voicu et al. [2] demonstrated that although pH-stat management resulted in higher intraoperative cerebral oxygen saturation levels, it did not confer a statistically significant survival benefit or reduction in long-term neurological deficits. These findings underscore that improving oxygen delivery alone does not guarantee improved neurological outcomes if autoregulatory mechanisms are disrupted. Instead, the preservation of stable cerebral perfusion pressures and controlled metabolic suppression, as achieved with α-stat, may offer superior neuroprotection by minimizing fluctuations in CBF and CMRO₂.

Collectively, the available evidence suggests that while pH-stat may provide certain intraoperative benefits related to oxygen delivery, α-stat offers a more physiologically stable approach that aligns better with the brain’s metabolic adaptations during hypothermia, particularly in adult patients.

Discussion

Findings from this systematic review indicate that the pH-stat strategy improves cerebral oxygenation in neonates and pediatric patients. This effect is primarily attributed to CO₂-induced vasodilation under hypothermic conditions, which enhances CBF distribution and promotes more rapid and uniform cerebral cooling. These mechanisms may be especially advantageous in neonates and infants whose immature autoregulatory systems render them more vulnerable to ischemic events during hypothermia. However, this benefit may be offset by impaired cerebral autoregulation and an increased risk of hyperemia or microembolism formation, particularly in adult patients undergoing prolonged aortic arch procedures [2,6,7].

In contrast, α-stat management preserves the physiologic decline in pH associated with hypothermia, thereby supporting cerebral autoregulation, stabilizing intracellular enzymatic activity, and allowing more controlled reductions in CMRO₂. Several studies associate α-stat with reduced incidence of postoperative neurological deficits in adults due to its ability to maintain stable PaCO₂ levels and prevent cerebral hyperperfusion during both cooling and rewarming phases [1,2,8,9]. These findings support the hypothesis that α-stat more closely mirrors the natural metabolic suppression that occurs under hypothermia, thus minimizing fluctuations in cerebral perfusion parameters and protecting neural tissue integrity.

Collectively, these physiological distinctions underscore the need for age- and context-specific acid-base strategies. Durandy [6] and Voicu et al. [2] proposed a hybrid approach utilizing pH-stat during the cooling phase to ensure uniform cerebral cooling and switching to α-stat during rewarming to preserve autoregulatory function. Likewise, Yu et al. [7] and Werner et al. [9] advocated for individualized strategies based on patient-specific cerebral risk factors, surgical duration, and target temperature.

The dynamic behavior of cerebral oxygenation indices such as SjvO₂ and CEO₂ under pH-stat suggests the potential for improved oxygen delivery in pediatric patients. Conversely, α-stat consistently maintains more stable suppression of CMRO₂ and may reduce neurologic injury in adult populations. These observations indicate that no single approach is universally superior; rather, acid-base management should be tailored to the individual clinical context [1,2,7,9].

**Figure 2:**
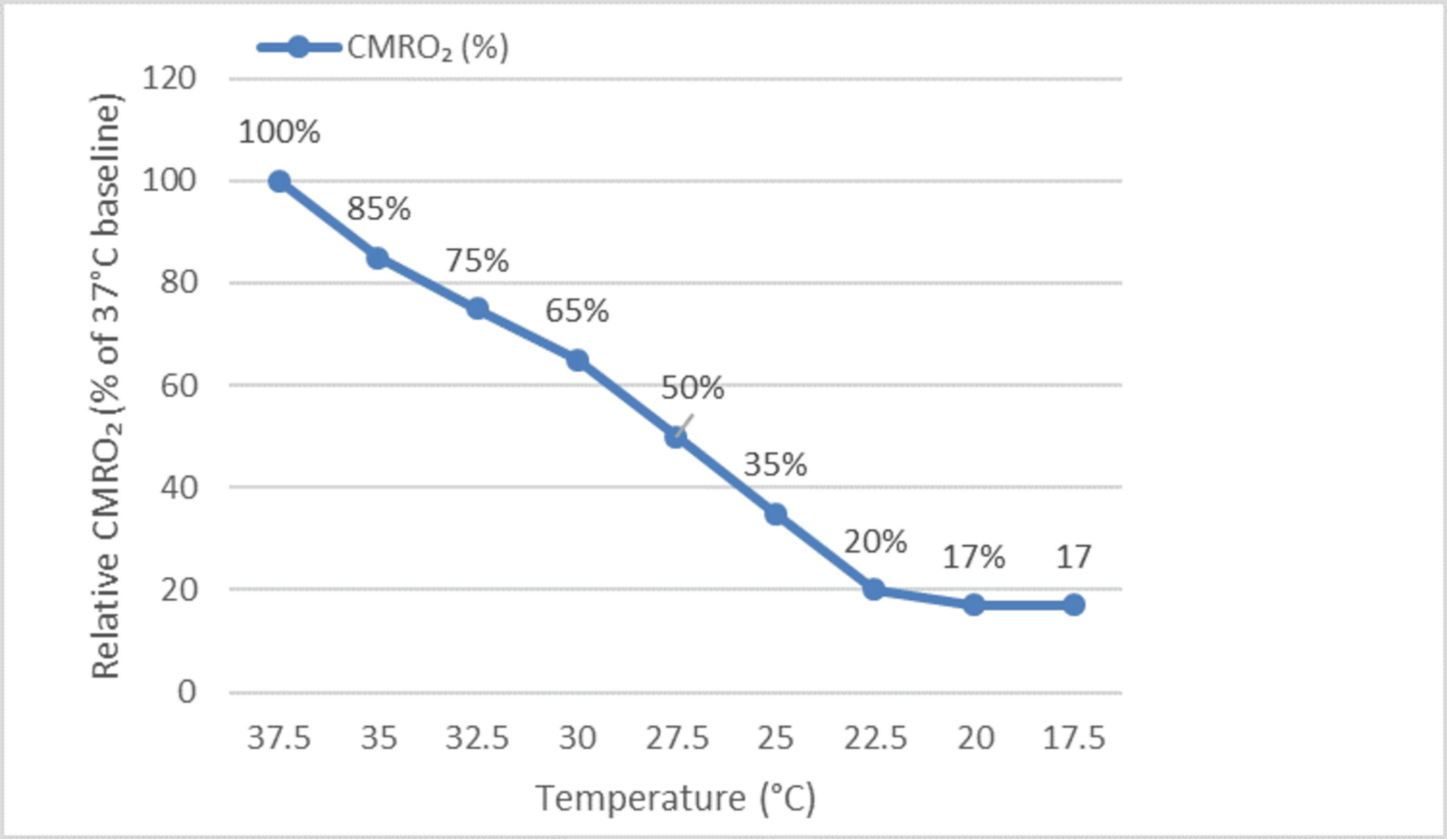
Relationship between cerebral metabolic rate of oxygen (CMRO₂) and temperature. Illustration created by the authors based on data from Svyatets et al. [10] and Durandy [6].

Figure 2 illustrates the inverse relationship between CMRO₂ and decreasing temperature.

Durandy [6] and Voicu et al. [2] support a dual-phase approach using pH-stat for induction and α-stat for rewarming. Meta-analyses by Yu et al. [7] and Werner et al. [9] further reinforce the need for individualized strategy selection based on patient age, procedural characteristics, and cerebral vulnerability.

**Figure 3:**
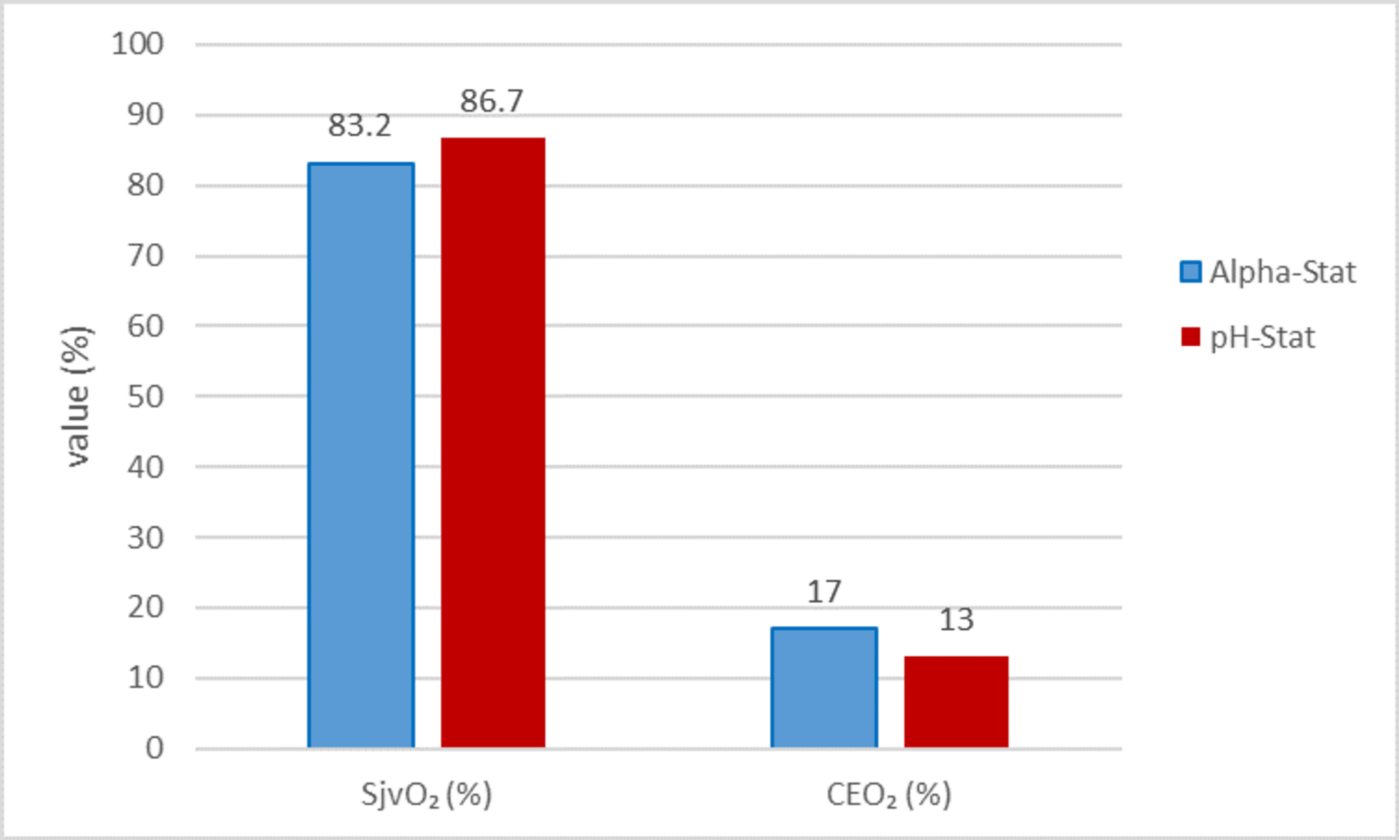
Cerebral oxygenation metrics: α-stat versus pH-stat. Comparison of SjvO₂, CEO₂, and AJDO₂ under α-stat and pH-stat blood gas management strategies during hypothermia. pH-stat demonstrated higher cerebral oxygen saturation and reduced oxygen extraction. Illustration created by the authors based on data from Voicu et al. [3].

The differences in cerebral oxygenation metrics between α-stat and pH-stat techniques are highlighted in Figure 3.

Clinical application of these findings should also consider institutional protocols, perfusionist expertise, and available monitoring technologies, as intraoperative variability can significantly influence outcomes [7,9].

**Figure 4:**
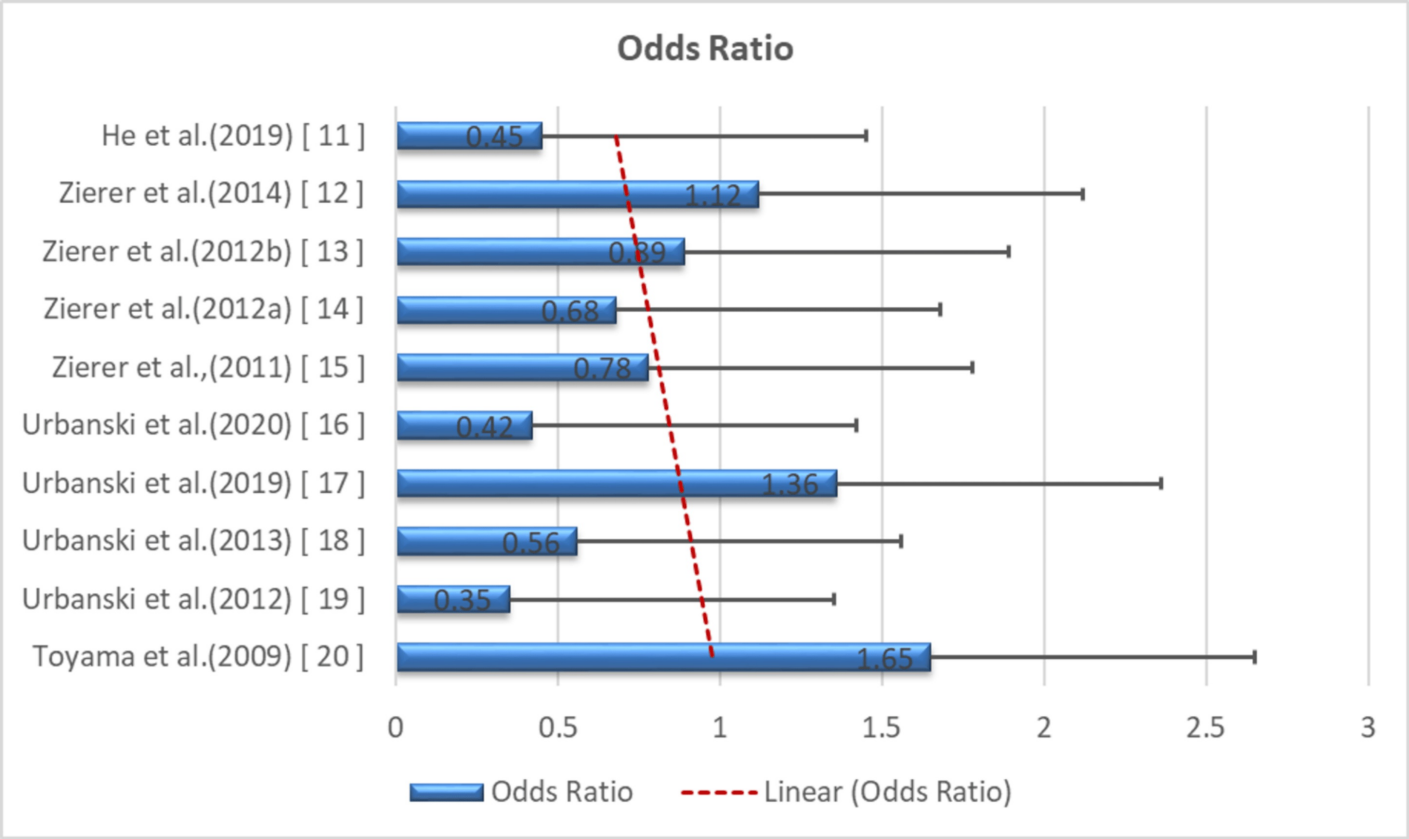
Forest plot of neurological outcome risk. Forest plot of neurological outcome risk comparing pH-stat and α-stat blood gas management. Odds ratios and 95% confidence intervals comparing neurological outcomes under pH-stat versus α-stat strategies are shown. The vertical dashed line indicates the null effect (odds ratio = 1.0). Data synthesized from included studies [11-20], based on Yu et al. [7].

Figure 4 presents pooled data from the included RCTs, demonstrating a consistent trend favoring the α-stat strategy in reducing adverse neurological outcomes [11-20].

**Figure 5:**
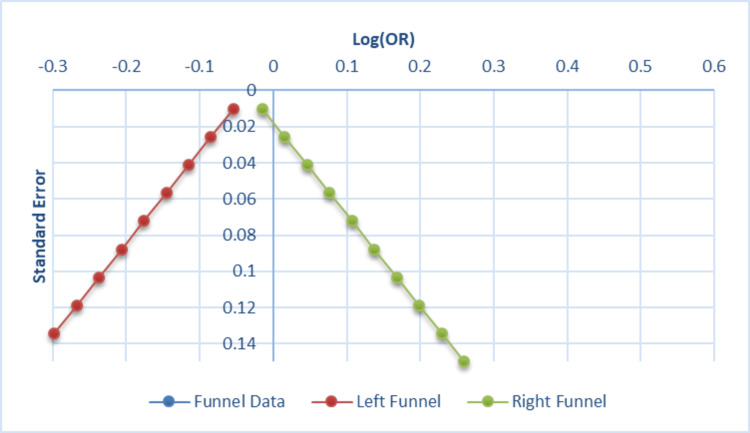
Funnel plot of publication bias. Funnel plot assessing publication bias based on the meta-analysis by Yu et al. [7]. Standard error is plotted against effect size (odds ratio) for each included study. The symmetry of the plot suggests a relatively low risk of publication bias.

Figure 5 displays the funnel plot from the meta-analysis by Yu et al. [7], assessing publication bias and study heterogeneity.

**Figure 6:**
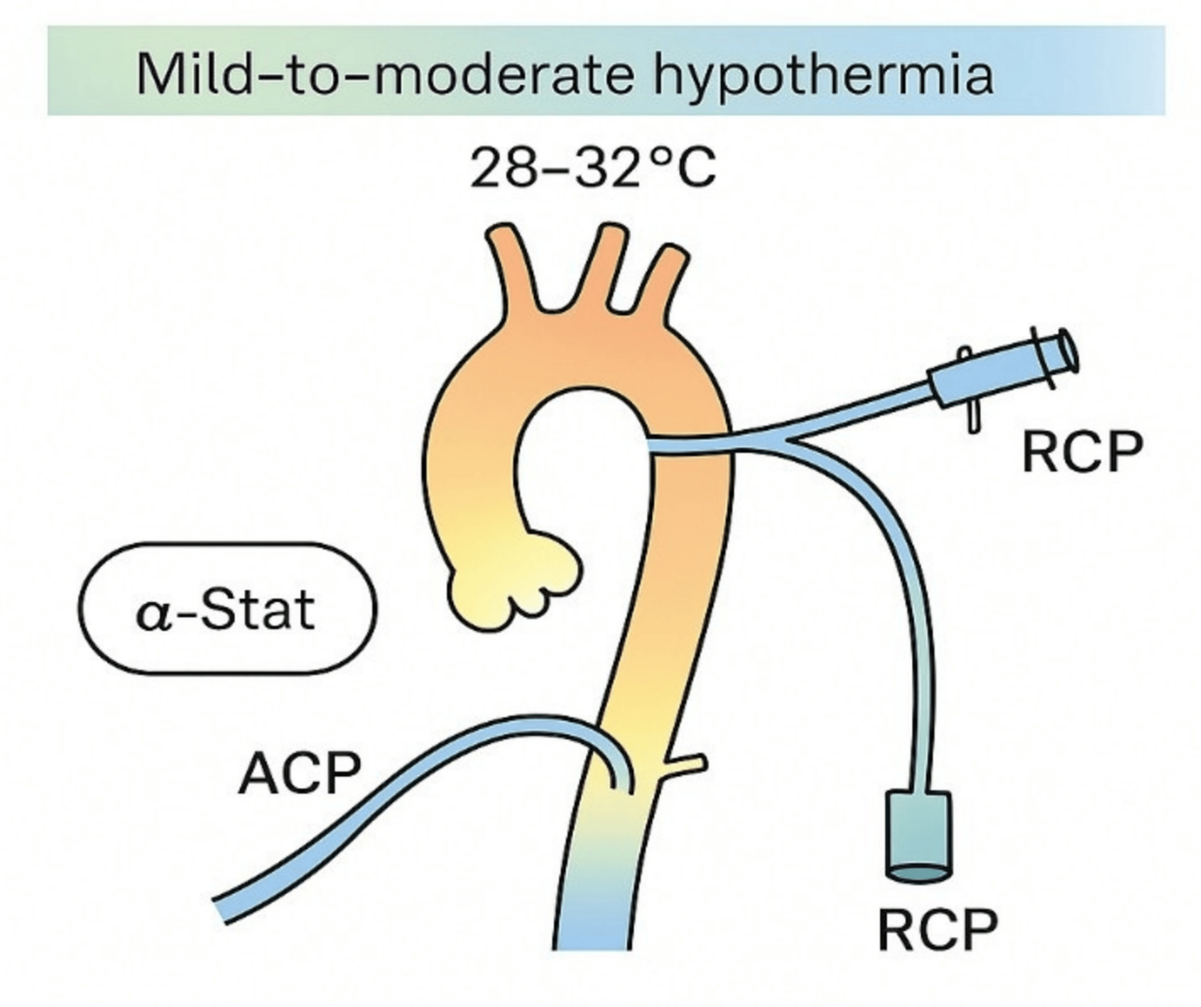
Vienna perfusion protocol. A schematic overview of the Vienna perfusion protocol utilizing moderate hypothermia (28–32°C), α-stat blood gas management, and combined antegrade and retrograde cerebral perfusion. The protocol avoids deep hypothermia to support cerebral autoregulation. Illustration created by the authors based on data from Werner et al. [9]. ACP: antegrade cerebral perfusion; RCP: retrograde cerebral perfusion

Figure 6 outlines the Vienna perfusion protocol, aligning with contemporary best practices in adult cerebral protection during moderate hypothermia [9].

Future research should investigate long-term neurodevelopmental outcomes in pediatric populations and cognitive trajectories in adult survivors following HCA. As intraoperative neuromonitoring technologies such as near-infrared spectroscopy and cerebral microdialysis continue to advance, real-time feedback may enable more precise titration of perfusion strategies. Standardizing outcome metrics and establishing evidence-based guidelines will require close interdisciplinary collaboration among cardiothoracic surgeons, neurologists, anesthesiologists, and perfusionists to optimize neuroprotection in diverse surgical contexts.

Limitations

This systematic review has several limitations that warrant consideration. The included studies demonstrated considerable heterogeneity in study design, patient populations, surgical techniques, cooling protocols, outcome measures, and follow-up duration, which complicates direct comparison and meta-analytic synthesis. Many of the RCTs had relatively small sample sizes, reducing statistical power and increasing the potential for type II error. Additionally, several studies focused primarily on surrogate markers such as cerebral oxygenation indices (SjvO₂, CEO₂), rather than clinically relevant long-term neurological or cognitive outcomes. The variability in neuroprotective monitoring modalities further limits the generalizability of the findings. Furthermore, language and publication bias may be present as the review was restricted to peer-reviewed, English-language articles. Finally, the absence of large-scale, multicenter randomized trials limits the ability to draw definitive conclusions regarding the superiority of one acid-base management strategy over the other across diverse surgical contexts.

## Conclusions

The results of this systematic review suggest that pH-stat management offers specific intraoperative advantages in pediatric and neonatal populations by enhancing cerebral oxygenation and facilitating more rapid induction of hypothermia. In contrast, α-stat management appears more appropriate for adult patients, as it better preserves cerebral autoregulation, maintains metabolic suppression, and is associated with lower rates of postoperative neurological complications. While hybrid strategies incorporating both approaches during distinct phases of cooling and rewarming may provide additional benefit, no single strategy has demonstrated universal superiority across all patient populations. Therefore, acid-base management during HCA should be individualized based on patient-specific factors, including age, cerebral vulnerability, surgical complexity, and institutional protocols, to optimize neuroprotection and procedural outcomes.
